# Curriculum of bachelor’s degree in forensic science at Al Istiqlal University in Palestine and students’ evaluation of the model

**DOI:** 10.1186/s41935-023-00335-4

**Published:** 2023-03-08

**Authors:** Walid M. Khalilia

**Affiliations:** Forensic Science Department, Al Istiqlal University, Jericho, Palestine

**Keywords:** Forensic science, Physical evidence, Crime scene, Curriculum, Al Istiqlal University, Justice system

## Abstract

**Background:**

Any science can be a forensic science (FS) if it has some justice application. Therefore, FS is an extremely interdisciplinary field at the interface of chemistry, biology, physics, medicine, law, criminology, and other related sciences. The Al Istiqlal University obtained the first and only license in Palestine to open a bachelor’s program specialized in FS, and there is a limited number of similar programs in the region, while there is growth around the world in this field. A full bachelor’s program in FS has been established at the Al Istiqlal University. This study aimed to describe the FS program model and its curricula and, in addition, evaluation of such a program from its students’ point of view. To achieve the aims of this study, a questionnaire was developed, and its validity and reliability were confirmed. The study sample consisted of all 35 male and female students enrolled in the FS program at the Al Istiqlal University for the spring semester of the academic years 2021–2022.

**Results:**

After collecting and analyzing the study tool, the results showed that the students agreed on all the paragraphs of the questionnaire that encourage the specialization of FS according to the model established at the Al Istiqlal University. The overall degree of students’ evaluation of the FS program was high, and the total mean of the students’ responses for all study fields was 3.56. The total degree for determining the students’ evaluation of faculty members, students’ evaluation of curricula and contents, and students’ evaluation of academic support services came to a high degree with a means of 3.75, 3.51, and 3.42 respectively.

**Conclusion:**

This study recommended the importance of the Bachelor of FS Program at the Al Istiqlal University and recommended developing disseminating and raising awareness of this new scientific field. The success of the Bachelor of FS program at the Al Istiqlal University is a model for the region to follow.

**Supplementary Information:**

The online version contains supplementary material available at 10.1186/s41935-023-00335-4.

## Background

Forensic science (FS) is the application of scientific principles and techniques to matters of criminal and civil law. It involves the observation, documentation, collection, analysis, assessment, and scientific interpretation of physical evidence. Today FS is one of the most important pieces of a case in modern law and one of the few areas of law enforcement where science, technology, and crime solving meet (Samarji 2010). This field of science is extremely wide field as any science or piece of information used to help with settling a legitimate issue or case can appreciate the descriptor “forensic” in such a context (Eckert William 1992; Robertson 2012).

FS utilizes physical, natural, clinical, and even behavioral sciences to examine, analyze, and evaluate all types of physical evidence, individuals like suspects and victims, and even trace evidence for example poisons in blood to issues relating to the law (Quarino and Brettell 2009; Saferstein and Roy 2021). Contrary to what some people may believe, FS is not just concerned with criminal issues and law. Its scene grows to cover civil laws and cases (Bell et al. 2008; Mateen and Tariq 2019).

FS practices include pattern evidence such as fingerprints, firearms examination, tool marks, bite marks, impressions like tires and footwear, bloodstain pattern analysis, handwriting, and hair. In addition, FS practices include analytical evidence such as coatings like chemicals, paint, materials including glass, deoxyribose nucleic acid (DNA), fluids, serology, and explosive analysis (National Research Council (NRC) (U.S.) 2009; Samarji 2010; Saferstein and Roy 2021).

Recently, FS has stood out as one of the fields of academic studies in hundreds of higher educational institutions worldwide offering FS programs (National Institute for Forensic Science (NIFS) 2006; Fairgrieve 2010). FS education has been marked by an exponential increase in the number of students enrolled in several programs offered around the world (Houck and Siege 2006; Mennell 2006).

The Al Istiqlal University is engaged in a prospective long-term strategy to create a highly specialized core group that satisfies the preferences and performance standards of work within Palestinian government entities. Moreover, it is judicial, social stability, and military agencies that can are able to engage in reasonable scientific and knowledgeable circumstances in all disciplines of work. It is striking to note that the FS program is the only one to offer a university degree in FS in Palestine. In fact, the Al Istiqlal University is exceptional in this initiative, which plays a significant role in meeting the demands of the workplace and preparing dedicated staff with a high level of expertise in forensic laboratory analysis and crime scene investigation (CSI) for crime investigative process that the security and judicial departments have to uncover factual data.

Its unique importance stems from the notion that the Department of FS at the Al Istiqlal University tackles a genuine gap in the Palestinian judicial system. This system lacks appropriately trained, talented staff. Therefore, the Al Istiqlal University is engaged in a long-term goal to build a highly qualified cadre that takes into account the requirements and standards of employment within the Palestinian legal system. Graduates from the university have the core expertise to serve in the areas of public litigation, the judicial system, and the judicial police (police and security services). Along with the military and civil court systems, other institutions also require such qualified personnel. This program would provide all of these institutions with qualified and specialized personnel who could handle forensic evidence in an appropriate, scientifically sound, and operationally reasonable manner. They could handle these materials while performing CSI tasks like collection, documentation, storage, and protection. Furthermore, through forensic lab activities and analysis, until the required technical expertise report was published and court evidence that would eventually pursue justice and defend freedoms and human rights.

On the other hand, the evaluation of the Bachelor of FS program at the Al Istiqlal University recommends a review of the program’s content, teaching competencies, and assessment procedures, which underlines the significance of this study, thus providing the academy leaders to receive feedback useful in improving and developing the content of the program, the methods of teaching and assessment followed, and communication between students and faculty members. Moreover, this study can benefit researchers in conducting evaluation studies from other aspects. The scientific importance of the study appears in that it is an attempt to enrich the educational library with educational literature related to evaluating academic programs within the framework of achieving total quality. The aim of this study is determined in evaluating the Bachelor of FS program in the Faculty of Law and FS at the Al Istiqlal University. To ensure that the Bachelor of FS program achieves its mission and objectives, it is necessary to review and evaluate this program, in terms of its content, methods of teaching, and assessment used in it and, moreover, communication between the members of the teaching and students from the student’s point of view, to ensure the effectiveness of this program.

## Methods

### Development of the FS program

**Fig. 1:**
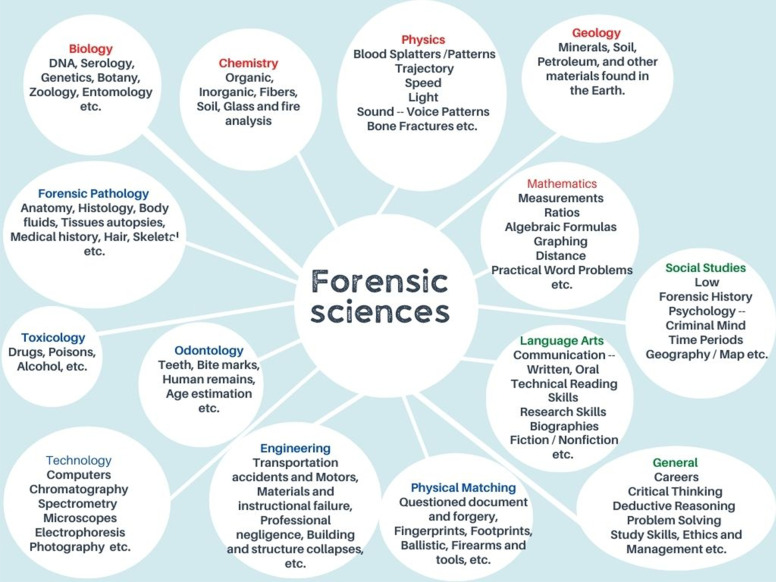
FS research and education fields

During the establishment of the bachelor program in FS, all areas of learning and research related to FS (Fig. 1) were searched. In addition, document analysis was conducted on the most published curricula of undergraduate FS programs offered throughout the world (Lucas and Sharpe 1969; Bari Lateef 1974; National Institute of Justice (NIJ) (U.S.) 2007; National Research Council (NRC) (U.S.) 2009; Fairgrieve 2010; Edmond et al. 2016). Data screening produced a comprehension of the status of FS instruction, a field where little is known or distributed. It likewise produced conceptual features about the knowledge base integrated within FS and the nature of the practice of FS and its identity.

At the Al Istiqlal University, several departments and institutes have research and educational activities related to FS. The Al Istiqlal University has several specialized instructors and research teams interested in studying and teaching types of physical forensic evidence, crime scene, criminal law, criminology, and other human sciences, in addition to specialists in biology, chemistry, toxicology, physics, mathematics, forensic medicine, and other natural sciences and their applications and, moreover, to information technology and data management teams that teach cybercrime and related topics.

The Al Istiqlal University has cooperation agreements with local universities to teach some practical courses specialized in its laboratories, such as forensic chemistry, forensic biology, and toxicology analyses. Additionally, there is an agreement between the Al Istiqlal University and the management of the several Palestinian forensic labs run by the Palestinian Police Agency, where the majority of the graduate students in this program will work. Thus, throughout their term at the university, those students can finish their graduation projects and receive instruction in the departments of these laboratories.

### Evaluation of the FS program

#### Study sample

The target group of this study consisted of all undergraduate students (*n* = 40) from the third and fourth study grades in the FS department at the Al Istiqlal University during the spring semester of 2021/2022.

#### Study tool

To achieve the aim of this study, an item-based questionnaire was developed to evaluate the Bachelor of FS program based on previous studies and online resources (Mennell 2006). The content validity of the initial questionnaire was measured through five peer reviewers who are faculty staff members responsible for learning in their universities. To ensure the reliability of the questionnaire, it was determined by internal consistency reliability (Cronbach’s *α* = 0.950). The study tool, in its final form, consisted of 51 paragraphs (Additional file 1: Appendix 1), from which personal and demographic variables (3 questions), three parts containing 48 closed questions items (5-point Likert scale), were grouped into students’ evaluation of faculty members (21 questions), evaluation of curricula and contents (14 questions), and evaluation of academic support services (13 questions). Access to the questionnaire was provided to all students (40) by an email link in March 2022. Thirty-five valid questionnaires were received from that sample.

### Statistical analysis

Students’ responses were unloaded, and appropriate statistical treatments were performed using the SPSS 19.0 statistics software to test the frequency distribution and mean reports. ANOVA variance analysis, *t*-test, and Pearson correlation coefficient were applied to test for statistical significances between various measures in this study, and mean values were used to perform principal component analysis (PCA) to test whether the variables are correlated in the population. The significance level was accepted at *P* < 0.05.

## Results

### Development of the FS program

Due to the interdisciplinary nature of the program, the Al Istiqlal University had to decide where to affiliate the bachelor program. Since CSI, criminology, forensic evidences, and forensic medicine are core disciplines in law, it was logical to associate the course with the Faculty of Law at the Al Istiqlal University. However, key expertise in biology and chemistry is associated with the other faculties. The location of a specialist Institute of Forensic Laboratories that is associated with the college of law and FS at Al Istiqlal University is significant. In the Faculty of Law and FS, there is a Department of Criminology and Law, which contributes to providing the program with many experts and related courses such as Criminology, Criminal Procedure Law, and the general Penal Code. Other relevant disciplines including Civil Law, Democracy and Human Rights, CSI, and Forensic Medicine are associated with the Department of Police Science and Low.

### FS curriculum

The plan of the FS bachelor program includes 147 required credit hours as a university, college, academic-military, and department specialization requirements. In addition to what is permitted in security academies and police colleges, all program courses throughout eight semesters are extracurricular requirements that include the development of core skills for security professionals. Detailed information on the full FS program can be found at https://fl.alistiqlal.edu.ps/page-322-ar.html

### Descriptive statistics of sample characteristics

To display the distribution of the study sample members according to its variables, the frequency and percentage were calculated according to the variables (gender, academic grade, and credit hours completed) (Table 1).

**Table 1:** Descriptive statistics of sample characteristics

Characteristics	Category	Frequency (***n*** = 35)	Percentage %
**Gender**	Male	25	71.4
	Female	10	28.6
**Credit hours completed**	Less than 121	16	36.7
	More than 121	19	54.3
**Academic Grade**	3rd grade	16	45.7
	4th grade	19	54.3

It is clear from the Table 1 that the distribution of the study sample according to gender was 71.4% males, while the percentage of females was 28.6%, as this percentage comes naturally because of the student admission criteria at the Al Istiqlal University and the nature of work in security agencies in Palestine, where the percentage of females does not exceed 30%.

In the distribution of the study sample according to the academic grade between the third and fourth levels, the highest percentage for the 4th grade was 54.3%, followed by the 3rd grade with a rate of 45.7%. This is normal because each level of the program has 20 students. In addition, the questionnaires that were excluded belonged to the 3rd grade students.

### Statistical descriptions of the students’ evaluation

Fifteen female and 25 male students were involved in this study because they had passed many credit hours (80 credit hours or more), and they have sufficient knowledge about the study plans, courses, and faculty members, which makes third and fourth-grade students able to evaluate the quality of academic programs more than first and second-grade students.

It is clear from Table 2 that the means and standard deviations generally indicate positive trends toward the FS program model and its curricula, where the number of paragraphs whose means were greater than the hypothetical average 3 is 47 paragraphs, while only one paragraph is less than 3, which indicates that the evaluation of students of the Department of FS at Al Istiqlal University was positive towards this model and its curricula.

**Table 2:** Descriptive statistical analysis of students’ evaluation of the FS program at the Al-Istiqlal University

#	Paragraph	Mean	Std.
1.	**Students’ evaluation of faculty members**		
1.	The semester plan is distributed to the students at the beginning of the semester	3.83	1.10
2.	The lecturer takes into account answering the students’ questions	4.09	0.56
3.	Students’ notes are worked out and feedback is taken	3.66	0.73
4.	The lecturer shall abide by the time specified for the lecture	3.74	0.78
5.	The lecturer sets the course requirements: exams, readings, and worksheets at the beginning of the semester	3.54	0.78
6.	The department in the college determines lecture times	4.23	0.55
7.	The lecturer presents the topic sequentially and logically	3.97	0.57
8.	The lecturer directs the students on how to obtain information sources	3.77	0.55
9.	The lecturer employs a variety of teaching resources	3.54	0.82
10.	The lecturer employs a variety of teaching methods	3.71	0.83
11.	The lecturer uses sound language and appropriate terminology that is easy to understand	4.03	0.62
12.	The lecturer works on brainstorming students’ ideas during lectures	3.80	0.47
13.	The lecturer motivates the students during class participation	3.83	0.75
14.	The lecturer uses various methods of discussion	3.69	0.68
15.	The lecturer collects the scientific material related to the topic of the lecture from multiple references	3.57	0.81
16.	The lecturer does modern pedagogical strategies during the semester	3.31	0.93
17.	The lecturer shows enthusiasm and vitality in the lecture	3.63	0.73
18.	The lecturer encourages students to think and be creative	3.77	0.77
19.	The lecturer takes into account the humanitarian conditions of the students during the lectures	3.74	0.78
20.	The lecturer cooperates with the military side in conducting the educational process	3.69	0.93
21.	The lecturer concludes the lecture by summarizing its highlights	3.60	0.95
**The overall score for the first field**		**3.75**	**0.39**
2.	**Students’ evaluation of curricula and contents**		
22.	Clarity of language in university teaching courses used	3.71	0.79
23.	The general and specific objectives of the curriculum are specified in the course units	3.60	0.81
24.	Correlation between general and specific objectives and the curriculum content	3.69	0.90
25.	Compatibility between technology and curricula	3.37	1.11
26.	Sequence of displaying information according to the course material	3.83	0.71
27.	Diversity in the evaluation methods used	3.63	0.73
28.	The relationship between students’ abilities, their learning environment, and the knowledge content of the subject	3.60	0.81
29.	The courses motivate the students	3.23	1.00
30.	Knowledge experiences fit the needs of society and students	3.49	0.95
31.	The proportion between the size of the lectures and the number of lectures scheduled for teaching	3.49	0.85
32.	The courses link theoretical and applied information	3.29	1.07
33.	The courses include activities that help critical thinking, inquiry, and analysis	3.26	0.92
34.	Courses are compatible with the requirements of the labor market	3.43	0.98
35.	The courses contain elements of attraction and suspense	3.51	0.92
**The overall score for the second field**		**3.51**	**0.62**
**3.**	**Students’ evaluation of academic support services**		
36.	The university library facilitates the teaching process and increases experiences	3.49	0.95
37.	The university library employs modern technology	3.60	0.91
38.	The university employs modern technology to facilitate students’ transactions and needs	3.31	1.02
39.	The university provides the appropriate environment to assist students in academic interaction	3.11	1.13
40.	The university has facilities to provide food to suit the needs of students	3.66	0.97
41.	The university provides psychological counseling centers for students and works to treat some cases	2.94	1.14
42.	The university administration works on developing student talents	3.43	1.04
43.	Health, security, and safety conditions are met in the university facilities	3.83	0.98
44.	The university attaches importance to extracurricular activities to deepen the relationship between students and faculty members	3.37	0.97
45.	The university has computer laboratories in proportion to the number of students	3.31	0.99
46.	The university buildings and their grounds are under constant maintenance	3.54	0.95
47.	The classrooms have the means and devices that facilitate the learning process	3.69	0.87
48.	The university provides private and comfortable rooms for students to rest in the department and college	3.23	1.33
**The overall score for the third field**		**3.42**	0.70
**The overall score for the three fields**		**3.56**	0.50

Table 2 shows that the means of all fields of study are high (3.56). The total degree for determining the students’ evaluation of faculty members, students’ evaluation of curricula and contents, and students’ evaluation of academic support services came to a high degree with a mean of 3.75, 3.51, and 3.42 respectively.

Table 2 indicates that the total score for the first field, “students’ evaluation of faculty members,” was significant, as the mean was 3.75, with a standard deviation of 0.39, i.e., a percentage of 75%. The results also show in this field that the paragraph “the department in the college determines lecture times” received the highest evaluation rate by students, with a percentage of 85%, while the paragraph “the lecturer does modern pedagogical strategies during the semester” was awarded the lowest evaluation rate by students, with a percentage of 66%.

The total score for the second field, “students’ assessment of the curricula and their contents” was significant, as the mean was 3.51, with a standard deviation of 0.62, i.e., a percentage of 70%. The results also show in this field that the paragraph “sequence in displaying information according to the course material” received the highest evaluation rate by students, with a percentage of 77%, while the paragraph “the courses motivate the students” has the lowest evaluation rate by students, which was a percentage of 65% (Table 2).

Table 2 also indicates that the total score for the third field, “students’ evaluation of academic support services,” was significant, as the mean was 3.42, with a standard deviation of 0.70, i.e., a percentage of 68%. The results also show in this field that the paragraph “health, security and safety conditions are met in the university” got the highest rate of assessment by students with a percentage of 77%, while the paragraph “the university provides psychological counseling centers for students and works to treat some case” has the lowest evaluation rate by students, which was 59%.

It is clear from Table 3 that there are no significant differences (*P* < 0.05) between the means of the responses of students according to gender in fields of the study tool.

**Table 3:** Means, standard deviations, one-way ANOVA analysis of students’ evaluation of the FS program at the Al-Istiqlal University according to gender

Evaluated issue	Gender**	Mean	Std.	***F***	(Sig)
Students’ evaluation of faculty members (teaching)	Male	3.747	0.331	− 0.025	0.980
	Female	3.752	0.545		
Students’ evaluation of curricula and contents	Male	3.466	0.609	− 0.633	0.531
	Female	3.614	0.675		
Students’ evaluation of academic support services	Male	3.419	0.691	− 0.076	0.940
	Female	3.439	0.748		
Total	Male	3.544	0.479	− 0.300	0.766
	Female	3.602	0.589		

*Statistically significant at *p* < 0.05; **Male (*n* = 25), female (*n* = 10)

It is clear from the results that there is a significant difference (*P* < 0.05) among the means of the responses according to the student’s academic grade and credit hours completed variables. It was found that the mean of students’ evaluation of faculty members and students’ evaluation of curricula and contents was significantly different in Tables 4 and 5.

**Table 4:** Means, standard deviations, and one-way ANOVA analysis of students’ evaluation of the FS program at the Al-Istiqlal University according to the academic grade

Evaluated issue	Academic grade	Mean	Std.	***F***	(Sig)*
Students’ evaluation of faculty members	3^rd^ grade (*n* = 16)	3.5893	0.33486	− 0.025	0.025*
	4^th^ grade (*n* = 19)	3.8847	0.39883		
Students’ evaluation of curricula and contents	3^rd^ grade (*n* = 16)	3.2143	0.66803	− 2.813	0.008*
	4^th^ grade (*n* = 19)	3.7556	0.46676		
Students’ evaluation of academic support services	3^rd^ grade (*n* = 16)	3.2837	0.53642	− 1.098	0.280
	4^th^ grade (*n* = 19)	3.5425	0.80289		
Total	3^rd^ grade (*n* = 16)	3.3624	0.447	− 2.258	0.031*
	4^th^ grade (*n* = 19)	3.7276	0.499		

*Statistically significant at *p* < 0.05

**Table 5:** Means, standard deviations, and one-way ANOVA analysis of students’ evaluation of the FS program at the Al-Istiqlal University according to the credit hours completed

	Credit hours completed	***N***	Mean	Std.	***F***	(Sig)
Students’ evaluation of faculty members	Less than 121	16	3.62	0.35	3.310	.049*
	More than 121	19	3.88	0.40		
Students’ evaluation of curricula and contents	Less than 121	16	3.28	0.68	5.140	.012*
	More than 121	19	3.76	0.47		
Students’ evaluation of academic support services	Less than 121	16	3.20	0.56	.608	.551
	More than 121	19	3.54	0.80		
Total	Less than 121	16	3.41	0.47	2.981	.065
	More than 121	19	3.73	0.50		

*Statistically significant at *p* < 0.05

To investigate the relationships among the items in the three evaluated issues, correlation matrix (Person) was determined in Table 6.

**Table 6:** Correlation matrix (Pearson) for the three evaluated issues

Evaluated issue	Faculty members	Curricula and contents	Academic support services
Faculty members	1	0.180	0.192
Curricula and contents	0.180	1	0.266
Academic support services	0.192	0.266	1

Table 6 shows that all item values are different from 0 with a significance level alpha = 0.05.

The least significant difference (LSD) test was employed for the dimensional comparison of means to determine who benefited from the differences (Dodge 2008). All differences were in favor of those students who were in the 4th grade and passed more than 121 credit hours (Table 7).

**Table 7:** LSD test of the principal components between the means of the credit hours passed

Evaluated issue	Number of credit hours passed	More than 121 credit hours	Less than 121 credit hours
Students’ evaluation of faculty members	Less than 121 credit hours	− .36090^*^	− 0.20952
	More than 121 credit hours		
Students’ evaluation of curricula and contents	Less than 121 credit hours	− .67772^*^	− 0.43636
	More than 121 credit hours		

### Principal component analysis (PCA)

**Fig. 2:**
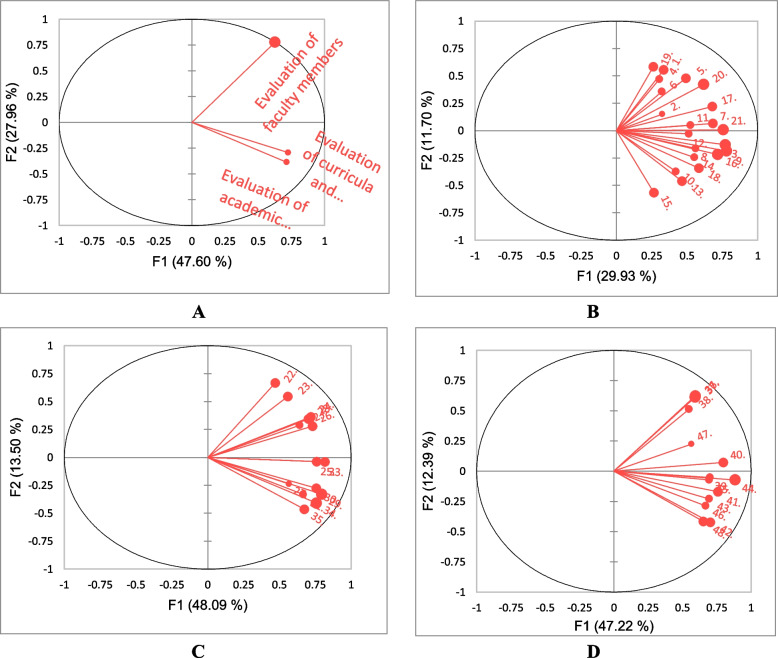
Graph of the correlations between the first two components of PCA and students answers regarding student’s grade. **A** Students’ evaluation of three variables. **B** Students’ evaluation of faculty members. **C** Students’ evaluation of curricula and contents. **D** Students’ evaluation of academic support services

Figure 2 presents the graphic representation of the correlations between the first two components (F1 and F2) and the student’s evaluation in PCA. F1 and F2 were associated with eigenvalues higher than one and accounted for 75.55% of the total cumulative variation: F1 47.60% and F2 27.96% of the total variability (Fig. 2A).

To extract the factors responsible for the discrepancies in answers between the third and fourth grades, PCA was run on each paragraph of the questionnaire (Fig. 2B–D).

Figure 2B presents the correlations between the first two components (F1 and F2) of students’ evaluation of faculty members in PCA: F1 29.93% and F2 11.70% of the total variability. It seems that there is a great weight of variations granted to the factors of the lecturer collecting the content of the lecture from multiple references, employing a variety of teaching methods, and motivating the students during class participation.

Figure 2C presents the correlations between the first two components (F1 and F2) of students’ evaluation of curricula and contents in PCA: F1 48.09% and F2 13.50% of the total variability. It seems that there is a great weight of variations granted to the factors of the courses containing elements of attraction and suspense and courses are compatible with the requirements of the labor market.

Figure 2D presents the correlations between the first two components (F1 and F2) of students’ evaluation of academic support services in PCA: F1 47.22% and F2 12.39% of the total variability. It seems that there is a great weight of variations granted to the factors of the university administration working on developing student talents and the university provides comfortable rooms for students to rest in the department and college.

## Discussion

A strong force for creating a representative government that considers human rights is FS (Crispino et al, 2014). Recently, FS has garnered interest on a global scale. Unfortunately, there is an upsurge in atrocities involving women and children, drug-related offenses, physical, sexual, and psychological maltreatment in Palestine. The Palestinian Central Bureau of Statistics (PCBs) launched the victimization survey in 2020 to track important criminal crime indicators in Palestine. In the country, 1.1% of people experienced criminal activity. The most common crimes committed against people were theft (57.3%), attempted robbery/theft (3.6%), assaults/threats (19.6%), property damage (4.8%), and harassment or assault by Israeli military or settlers (11.8%) (Palestinian Central Bureau of Statistics (PCBS) 2020). Figures prove that the Palestinian legal system is now processing a sizable number of criminal cases that must be handled with the production of convincing evidence. The Palestinian legal system likewise lacks appropriately qualified CSI employees. However, CSI findings can be easily misconstrued or even exploited against their intended audience, including lawyers, judges, the police, and politicians. Under these conditions, the university is required to act as a consultant and a credible source of criticism in Palestine about the application of scientific techniques for security, which it is required to oversee at the risk of harming the State of Law’s reputation.

To gain a better knowledge of the current academic condition of FS, documents found in the curricula of numerous programs have been examined (Additional file 1: Appendix 2). There is not much information or literature available on this area of education. To build a Bachelor of FS program in Palestine, it is also necessary to analyze the state of FS education and gain insights into what is known about FS. This supports Samarji’s earlier research, which was undertaken (Samarji 2010).

Since this specialization encompasses a wide range of theoretical and practical themes and its scientific justification is challenging, the researcher discovered from the review of FS programs that program designers confronted certain challenges in the building of the program. This is following other research that showed how intricate the field of FS is, utilizing techniques like DNA analysis, chemical composition, and pattern recognition (Bell et al. 2018).

The current study evaluated how well the newly developed FS program at the Al Istiqlal University was received by the students. The findings of this study show that students had a positive overall opinion of the FS program. The results indicate that the total score for students’ evaluation was significant, with a mean of 3.56, and a standard deviation of 0.50, i.e., a percentage of 71%. The first field, “students’ evaluation of faculty members,” received the highest evaluation, followed by the second field, “students’ evaluation of curricula and content,” and finally the third field, “student evaluation of academic support services” (Table 2). This is consistent with Thomas Gluodenis’ earlier research (Gluodenis 2021).

Male and female students at the Al Istiqlal University share identical living arrangements. All students are subject to the same training and class schedules, as well as common sleeping and lunchtimes because the institution is a security and military facility. Therefore, the rating of males and females did not differ significantly.

In terms of academic performance and credits obtained, students in the fourth academic year have finished more than 120 credits of the program, exhibiting their increased knowledge and skill with the study technique and subject matter. They were also taught by the bulk of the program’s instructors, who had privy to the vast majority of the university’s academic support services. As a result, the fourth-year students’ responses had higher averages than the third-year students.

The entire data set was treated to PCA to provide a thorough perspective of how the student evaluated the FS model concerning their grade. Using PCA, it was determined that the first two principal components (F1 and F2) accounted for 75.55% of the cumulative variation overall and were linked to eigenvalues greater than one. Most of the questionnaire’s paragraphs (> 0.5) have a substantial positive correlation with F1 (47.60%), while the instructor employs contemporary instructional techniques throughout the semester, paragraph 16 is inversely connected with this primary component. The student responses to paragraphs 1–4, 7, 10, 13–15, 22–27, 33, and 38–40 have a favorable impact on F2 (27.96% of the overall variability) (Table 2). It seems that there is a great weight of variations granted to the teaching staff dimension. Knowledge of the relationship between curriculum and instruction may help policymakers in the university to solve this problem and provide the desired quality education to students.

In the academic year 2019–2020, third graders began attending the university. This was also the start of the university’s tendency toward e-learning as a result of the COVID-19 pandemic, which lasted for almost 2 years. e-learning initially had several issues for both professors and students (Khalilia Walid 2020). It caused discrepancies in the way the students responded to the questionnaire’s sections on instruction, curriculum content, and university services. Whereas the third-year students, who were subjected to more e-learning, gave more negative responses, the fourth-year students’ responses were more favorable.

## Conclusions

A special bachelor’s degree at the Al Istiqlal University has been created to instruct high school students in the science-based branch in all pertinent FS topics. The objective is to educate undergraduates who are knowledgeable in a variety of topics and skills connected to the main theme FS and to address the severe lack of forensic specialists in Palestine, to conduct FS research, and to make Palestine a safer place to live. Although we expect our students to be knowledgeable in every aspect of FSs, they can select specific modules for their areas of interest, such as CSI, forensic biology, or forensic chemistry. The graduates should be fully conversant with all forms of forensic physical evidence; they should also be able to serve as expert witnesses who can testify admissible in court. They should also be proficient in forensic laboratory analysis and CSI for criminal investigations, which are needed by security and judicial agencies to find evidence and solve crimes.

The Al Istiqlal University developed the first bachelor’s program in FS in Palestine. This program can be used as a fresh model that other academic institutions in the area could implement. The findings of this study clearly show that the students had a positive overall opinion of the program.

## Supplementary Information

**Additional file 1: Appendix 1.** Questionnaire. **Appendix 2.** Best Universities in USA offering a Bachelor degree in forensic science.

## Data Availability

The datasets generated and/or analyzed during the current study are available in the PASS repository, https://fl.alistiqlal.edu.ps/page-322-ar.html. The datasets used and/or analyzed during the current study are available from the corresponding author on reasonable request.

## Abbreviations

CSI: Crime scene investigation
FS: Forensic science
DNA: Deoxyribose nucleic acid
