# Supplementary material for: Curriculum of bachelor’s degree in forensic science at Al Istiqlal University in Palestine and students’ evaluation of the model
Source: Egypt J Forensic Sci. 2023 Mar 8;13(1):16. doi: 10.1186/s41935-023-00335-4 (PMC9992912; doi:10.1186/s41935-023-00335-4)
Supplement: Supplementary file 1 — Additional file 1: Appendix 1. Questionnaire. Appendix 2. Best Universities in USA offering a Bachelor degree in forensic science. [file 41935_2023_335_MOESM1_ESM.docx]

**Appendix 1. Questionnaire**

Curriculum of Bachelor Degree in Forensic Science at Al-Istiqlal University in Palestine, and Students’ Evaluation of the Model.

1. **Students' evaluation paragraphs:**

| **#** | **Paragraph** | **Strongly agree** | **Agree** | **Medium** | **Disagree** | **Strongly disagree** |
| --- | --- | --- | --- | --- | --- | --- |
| **Students' evaluation of faculty members** | | | | | | |
|  | The semester course outline is distributed to the students at the beginning of the semester. |  |  |  |  |  |
|  | The lecturer takes into account answering the students' questions. |  |  |  |  |  |
|  | Students' notes are worked out and feedback is obtained. |  |  |  |  |  |
|  | The lecturer shall abide by the time allotted for the lecture. |  |  |  |  |  |
|  | The lecturer sets the requirements of the course: exams, readings, and worksheets at the beginning of the semester. |  |  |  |  |  |
|  | The department in the college determines lecture times. |  |  |  |  |  |
|  | The lecturer presents the topic sequentially and logically. |  |  |  |  |  |
|  | The lecturer directs the students on how to obtain sources of information |  |  |  |  |  |
|  | The lecturer employs a variety of teaching resources. |  |  |  |  |  |
|  | The lecturer employs a variety of teaching methods. |  |  |  |  |  |
|  | The lecturer uses sound language and appropriate terminology that is easy to understand. |  |  |  |  |  |
|  | The lecturer encourages students to brainstorm ideas during lectures. |  |  |  |  |  |
|  | The lecturer motivates the students to participate during class. |  |  |  |  |  |
|  | The lecturer uses various modes of discussion |  |  |  |  |  |
|  | The lecturer collects the scientific material related to the topic of the lecture from various references. |  |  |  |  |  |
|  | The lecturer does modern pedagogical strategies during the semester. |  |  |  |  |  |
|  | The lecturer exhibits enthusiasm and vitality in the lecture. |  |  |  |  |  |
|  | The lecturer encourages students to think and to be creative |  |  |  |  |  |
|  | The lecturer takes into account the human conditions of the students during the lectures. |  |  |  |  |  |
|  | The lecturer cooperates with the military side in conducting the educational process. |  |  |  |  |  |
|  | The lecturer sums up the lecture by providing summaries and highlighted ideas for it. |  |  |  |  |  |
| **Students' evaluation of curricula and contents** | | | | | | |
|  | Clarity of language in teaching university courses is used |  |  |  |  |  |
|  | The general and specific objectives of the curriculum are specified within the course units. |  |  |  |  |  |
|  | Correlation between general and specific objectives and the curriculum content. |  |  |  |  |  |
|  | Compatibility between technology and curricula. |  |  |  |  |  |
|  | Sequence in displaying information according to the course material (requirements). |  |  |  |  |  |
|  | Diversity in the methods of evaluation used. |  |  |  |  |  |
|  | The relationship between students' abilities, their learning environment, and the knowledge content of the subject. |  |  |  |  |  |
|  | The courses motivate the students. |  |  |  |  |  |
|  | Experiences in knowledge fit the needs of society and students. |  |  |  |  |  |
|  | The proportion between the size of the lectures and the number of lectures scheduled for teaching. |  |  |  |  |  |
|  | The courses link theoretical and applied information. |  |  |  |  |  |
|  | The courses include activities that help critical thinking, inquiry and analysis. |  |  |  |  |  |
|  | Courses are compatible with the requirements of the labor market. |  |  |  |  |  |
|  | The courses contain elements of attraction and suspense. |  |  |  |  |  |
| **Students' evaluation of academic support services** | | | | | | |
|  | The university library facilitates the teaching process and increases experiences. |  |  |  |  |  |
|  | The university library employs modern technology. |  |  |  |  |  |
|  | The university employs modern technology to facilitate students' transactions and needs. |  |  |  |  |  |
|  | The university provides the appropriate environment to assist students in academic interaction. |  |  |  |  |  |
|  | The university has facilities to provide food to suit the needs of students. |  |  |  |  |  |
|  | The university provides psychological counseling centers for students and offer treatment for some cases. |  |  |  |  |  |
|  | The university administration patronages students with talents. |  |  |  |  |  |
|  | Health, security and safety conditions are met in the university facilities |  |  |  |  |  |
|  | The university attaches importance to extracurricular activities to deepen the relationship between students and faculty members. |  |  |  |  |  |
|  | The university has computer laboratories in proportion to the number of students. |  |  |  |  |  |
|  | The university buildings and their grounds are under constant maintenance. |  |  |  |  |  |
|  | The classrooms have the means and devices that facilitate the learning process. |  |  |  |  |  |
|  | The university provides private and comfortable rooms for students to rest in the department and college. |  |  |  |  |  |

**II. Demographic Parameters:**

1. Gender: Male ( ) Female ( )
2. Study level: 3rd grade ( ) 4th grade ( )
3. Credit hours passed: Less than 121( ) More than 121( )

**Appendix 2. Best Universities in USA offering a Bachelor degree in forensic science.**

| **No.** | **University** | **BS Degree** | **Website** |
| --- | --- | --- | --- |
|  | Texas A&M University | Forensic and Investigative Sciences | <https://entomology.tamu.edu/forensic-investigative-sciences/> |
|  | San Jose State University | Forensic Science | <https://www.sjsu.edu/justicestudies/degrees/undergraduate-programs/bs-forensic-science/fs_major/index.html> |
|  | Cedarville University | Forensic Science | <https://www.cedarville.edu/academic-programs/forensic-science> |
|  | Ohio Northern University | Forensic Biology | <https://www.onu.edu/academics/forensic-biology> |
|  | University of Nebraska | Forensic Science | <https://catalog.unl.edu/undergraduate/agricultural-sciences-natural-resources/forensic-science/> |
|  | University of Central Florida | Forensic Science | <https://www.ucf.edu/degree/forensic-science-bs/> |
|  | Roger Williams University | Forensic Science | <https://www.rwu.edu/undergraduate/academics/programs/forensic-science> |
|  | Penn State University | Forensic Science | <https://science.psu.edu/bmb/forensics> |
|  | University of North Dakota | [Forensic Science](https://catalog.towson.edu/undergraduate/fisher-science-mathematics/chemistry/forensic-science/) | <https://und.edu/programs/forensic-science-bs/index.html> |
|  | George Washington University | Forensic Chemistry | <https://chemistry.columbian.gwu.edu/combined-bsmfs-forensic-chemistry> |
|  | West Virginia University | Forensic and Investigative Science | <https://admissions.wvu.edu/academics/majors/forensic-and-investigative-science> |
|  | Loyola University Chicago | Forensic Science | <https://www.luc.edu/forensicscience/bs.shtml> |
|  | University of Tampa | Forensic Science | <https://www.bachelorsdegreecenter.org/best-forensic-science-degree/> |
|  | Alfred State | Forensic Science Technology | <http://catalog.alfredstate.edu/current/programs/forensic-science-technology/> |
|  | University of Central Oklahoma | Forensic Science | <https://www.uco.edu/> |
